# Is novel research worth doing? Evidence from peer review at 49 journals

**DOI:** 10.1073/pnas.2118046119

**Published:** 2022-11-17

**Authors:** Misha Teplitskiy, Hao Peng, Andrea Blasco, Karim R. Lakhani

**Affiliations:** ^a^School of Information, University of Michigan, Ann Arbor, MI 48109;; ^b^Laboratory for Innovation Science at Harvard, Harvard University, Cambridge, MA 02138;; ^c^Harvard Business School, Boston, MA 02163;; ^d^Digital, Data and Design Institute, Harvard University, Cambridge, MA 02138

**Keywords:** novelty, peer review, bias, publishing

## Abstract

There are long-standing concerns that peer review, which is foundational to scientific institutions like journals and funding agencies, favors conservative ideas over novel ones. We investigate the association between novelty and the acceptance of manuscripts submitted to a large sample of scientific journals. The data cover 20,538 manuscripts submitted between 2013 and 2018 to the journals *Cell* and *Cell Reports* and 6,785 manuscripts submitted in 2018 to 47 journals published by the *Institute of Physics Publishing*. Following previous work that found that a balance of novel and conventional ideas predicts citation impact, we measure the novelty and conventionality of manuscripts by the atypicality of combinations of journals in their reference lists, taking the 90th percentile most atypical combination as “novelty” and the 50th percentile as “conventionality.” We find that higher novelty is consistently associated with higher acceptance; submissions in the top novelty quintile are 6.5 percentage points more likely than bottom quintile ones to get accepted. Higher conventionality is also associated with acceptance (+16.3% top–bottom quintile difference). Disagreement among peer reviewers was not systematically related to submission novelty or conventionality, and editors select strongly for novelty even conditional on reviewers' recommendations (+7.0% top–bottom quintile difference). Manuscripts exhibiting higher novelty were more highly cited. Overall, the findings suggest that journal peer review favors novel research that is well situated in the existing literature, incentivizing exploration in science and challenging the view that peer review is inherently antinovelty.

## Significance

There are long-standing concerns that scientific institutions, which often rely on peer review to select the best projects, tend to select conservative ones and thereby discourage novel research. Using peer review data from 49 journals in the life and physical sciences, we examined whether less novel manuscripts were likelier to be accepted for publication. Measuring the novelty of manuscripts as atypical combinations of journals in their reference lists, we found no evidence of conservatism. Across journals, more novel manuscripts were more likely to be accepted, even when peer reviewers were similarly enthusiastic. The findings suggest that peer review is not inherently conservative, and help explain why researchers continue to do novel work.

Much of modern science occurs within institutions; universities house researchers, funding agencies fund their work, and journals publish their findings. By allocating resources like money and attention, the institutions set the incentives that help determine which projects researchers pursue (2, 3). Projects that the institutions choose not to fund or publish are less likely to see the light of day. For example, between one-third and one-half of proposals rejected for funding and their associated research lines are subsequently discontinued by their authors (4).

A long-standing debate concerns whether these institutions incentivize projects that explore new knowledge terrain or conservative projects that make only incremental advances (5–9) and whether the current level of exploration is optimal (10, 11). The preferences expressed in various mission statements and stated criteria for selection are clear: institutions ubiquitously express support for novelty. For example, the US NIH seeks to “expand the knowledge base” and “foster fundamental creative discoveries [and] innovative research strategies” (12), while the journal *Nature* selects manuscripts in part on whether the “results seem novel, arresting (illuminating, unexpected or surprising)” (13).

Yet, accounts from many insiders paint a different picture (14, 15). The former NIH director Raynard S. Kington stated, “The system probably provides disincentives to funding really transformative research,” while the chief medical officer of the American Cancer Society Dr. Brawley admitted, “The problem in science is that the way you get ahead is by staying within narrow parameters and doing what other people are doing … No one wants to fund wild new ideas” (16). In the context of publishing, some authors report that papers that challenge existing perspectives face disproportionate criticism and are, therefore, harder to publish (17). Supporting this view, a large-scale analysis of academic publications showed that novel research had delayed recognition in terms of citations and was published in relatively low impact factor journals (18).

Adjudicating between mission statements and insider accounts is important not only for understanding what types of projects researchers are actually incentivized to pursue at various stages of the research pipeline, but also for administrators to identify organizational levers that can shift these incentives when needed (19, 20).

This paper has two aims. First, it aims to systematically identify the incentives for novel vs. conservative research in a particularly important context—publishing. Put simply, are novel papers harder to publish? The journal in which a paper appears continues to be the key signal of its importance (21), and in many fields, publications in a small set of top outlets are nearly required for jobs and promotions (22). Consequently, what such outlets deem publishable sends a strong signal to authors as they choose among projects to pursue.

We address this first aim using the review files of manuscripts submitted to a sample of 49 peer-reviewed academic journals. These data were obtained through separate agreements with the publishing companies Elsevier and the Institute of Physics Publishing (IOP) (*Data and Methods*). The *Elsevier* data cover 20,538 manuscripts submitted between 2013 and 2018 to the journals *Cell* and *Cell Reports,* both of which are published by Elsevier's subsidiary *Cell Press* and focus on the life sciences. The *IOP* data cover 6,785 manuscripts submitted in 2018 to its 47 physical sciences journals. In our analyses, we pool these datasets together into the “pooled dataset” and analyze them separately as “*IOP*,” “*Cell*,” and “*Cell Reports*.”

These data help overcome a key challenge of previous efforts to identify what journals select for: selection bias and difficulties of measurement. Many scientific organizations allocate resources using secretive processes of peer review. Analysts can typically observe the winning projects and papers but not the ones the institutions rejected. The several exceptions to this pattern consist of studies of funding competitions, and many conclude that the funders tend to favor conservative projects (10, 23–25) and scientists (26). For example, in one study biomedical scientists applying for seed grants with very novel ideas were less likely to get funding than those with moderately novel ideas, despite having similar track records (27). However, it is unclear if these findings generalize to other contexts since funding competitions involve highly uncertain ideas and tend to rely on researchers’ track records. In contrast, publishing involves assessing projects that are more complete, and researchers’ track records matter less. Studies of publishing tend to use published works only, usually finding that more novel works achieve higher citation impact (18, 28, 29). The citation premium suggests that novel work is rewarded by the scientific community (30), although there may be a long delay in realizing the premium (31), and some have argued that even the fully realized premium may not compensate for the increased risk (5). However, by not analyzing rejections such studies leave open the possibility that novel work is actually disfavored by journals and only novel work of unusually high quality is published (32).

Another challenge is measuring the novelty of projects objectively and at scale, while also accounting for their quality. Analysts typically focus on case studies of papers that prove to be novel over time (33–35). Such accounts tend to reach conclusions of conservatism. For example, Enrico Fermi’s seminal paper on weak interaction, one of the five fundamental forces of nature, was rejected from *Nature* for being “too removed from reality.” With the benefit of hindsight, such cases can be clearly called mistakes. Are these mistakes systematic? We address the measurement challenge by using recent advances in measuring novelty using references embedded inside of manuscripts (29).

The second aim of the paper is to illuminate the mechanisms by which pro- or antinovelty selections arise. We consider both “supply-side” mechanisms related to characteristics of the submission pool and “demand-side” mechanisms related to reviewers and editors’ choices. First, the supply of novel and conservative manuscripts may vary on their average level of quality. For example, if novel manuscripts are of lower overall quality, then there is likely a negative association between novelty and acceptance. Measuring scientific “quality” directly is notoriously difficult. Instead, we rely on two proxies—reviewer recommendations and citation impact—and relate both to novelty. On the demand side, evaluators may be affected by cognitive, strategic, or other forms of bias (24, 36). New ideas fit existing cognitive schemas imperfectly, generating uncertainty (27, 37, 38) and cognitive load (39). Evaluators may then reduce uncertainty by favoring conservative ideas (40, 41). Evaluators may also favor conservative ideas for a strategic reason, because those are less likely to displace their own “incumbent” ideas (36) Additionally, novel works may have higher variance in outcomes, and failures can come with outsized costs (e.g., reputational, political, etc.) relative to the rewards of success (42). Group discussions among editors may also increase conservatism (43). Novel work may induce more disagreement among reviewers, and if consensus is a near requirement for publication, the outcome may be lower odds of acceptance. All these mechanisms would lead more novel manuscripts to receive more negative evaluations. On the other hand, the cognitive burden of making sense of novel ideas may be lower when those are well connected to or “framed with” existing, well-understood ideas. If so, cognitively constrained evaluators may favor works that balance novelty and conventionality over those that lack one or the other (29). Lastly, recent work by Gross and Bergstrom (44) argues that novel ideas may be selected for when their producers have a mechanism (i.e., the results of their research) by which to persuade appropriately skeptical evaluators that the ideas are true. We return to this theory in *Conclusion*.

We address these mechanisms by measuring manuscript conventionality in addition to novelty, and measuring their associations with reviewer disagreement, reviewers’ recommendations, editors’ decisions, and citation impact.

Lastly, we investigate the role of editor discretion, which we define as selecting for or against novelty among manuscripts of similar quality. Previous research has consistently found that editors and other decision-makers broadly follow the recommendations of external reviewers but with occasional deviations (45–47). The nature of these deviations is poorly understood, although some work points to the role of author gender (48, 49) and correcting reviewer mistakes (50). We test whether these deviations are also explained by preferences for or against novelty by focusing on editors’ choices on papers receiving equally enthusiastic recommendations from peer reviewers.

Critical to our research question and design is the identification of a theoretically sound, reliable, and replicable quantitative measure of manuscript novelty. Following a long theoretical and empirical tradition in innovation research (51–55), we conceptualize the innovation process as recombining discrete components of existing knowledge. The combinations of components may be atypical (i.e., rarely done by previous researchers), in which case we consider them novel (28, 29, 51), or if those same elements have been frequently combined by others in the past, we consider them less novel. Although works such as patents or scientific papers can in principle consist of only one combination, in practice, they often contain several components (27), and each pair can represent a different level of novelty. Innovation researchers have consistently found that works that balance novel and conventional elements achieve greater success than ones containing primarily one type or the other (27, 29). In the context of combinations, such balance would be reflected by some combinations being very novel and others being very conventional.

We operationalize these intuitions in the context of academic publishing by defining “components of existing knowledge” as journals and their “combination” as appearing together in the list of references. For each pair of coreferenced journals, we assign a level of atypicality, reflecting how often have these two journals been combined by others in the existing literature. Specifically, we calculate a *z-*score for each journal pair that measures the degree to which the combination is atypical relative to a simulated null distribution of random journal-to-journal pairings (*SI Appendix*, *Novelty Measure* has details). This process yields for each manuscript a distribution of *z-*scores, one for each possible journal pair. Following ref. 29, we define “novelty” as the 10th percentile of this distribution of *z-*scores, which is the part of the distribution likely to contain the paper’s most novel contributions. Additionally, we define “conventionality” as the 50th percentile *z-*score from this distribution, reflecting how well the mass of the distribution is embedded in the existing literature. For interpretability, we reverse code novelty (but not conventionality), so that higher values indicate higher novelty. Lastly, we convert novelty and conventionality *z*-scores into percentiles, calculated separately for manuscripts in the life sciences and physics datasets, yielding final conventionality and novelty measures that range from 0 to 100, where a novelty of 100 denotes a manuscript with many extremely novel journal combinations and a conventionality of 100 denotes one where the bulk of combinations is extremely common. We distinguish this measure from quality and other potential confounds using additional controls (*Data and Methods* and Table 1). Specifically, we proxy quality primarily with reviewer recommendations and secondarily with citations.

**Table 1.** Variables used in life sciences and physics regressions

Variable	Description and notes	*Cell*	*Cell Reports*	*IOP*
Novelty	10th percentile most atypical journal pair among the pairs in a paper’s reference list converted to percentiles by ranking all values in either the life sciences or physics datasets and recoded to 100 = most atypical and 0 = least atypical	x	x	x
Conventionality	50th percentile most atypical journal pair in a paper’s reference list converted to percentiles by ranking all values in either the life sciences or physics datasets; 100 = most conventional, and 0 = least conventional	x	x	x
Year submitted	Some years of submission for *Cell Reports* were missing and imputed using year of publication –1 (*Data and Methods* has details)	x	x	x
No. of unique journals referenced	No. of unique journals referenced in a manuscript is divided by the total no. of references and standardized; controlling for this variable helps ensure that our results are not confounded by a mechanical relationship between novelty and the number of unique journals referenced	x	x	x
No. of authors		x	x	x
No. of references		x	x	x
Last author no. of publications	Proxy for author status and experience up to submission year (publication year for *Cell Reports*)	x	x	
Last author no. of publications in *Cell* or *Cell Reports*	Proxy for authors’ knowledge of the journals and editors’ knowledge of the authors calculated up to submission year (publication year for *Cell Reports*)	x	x	
No. of references to *Cell* or *Cell Reports*	Proxy for the authors’ knowledge of journal scope and the journal’s prior interest in the submission topic	x	x	
Citations after {1 or 5} y	Inclusive of citations received in the year of publication	x	x	x
Reviewer enthusiasm	Mean recommendation by external reviewers in the first review round; recommendations were coded {1 = accept or revise and resubmit, 0 = reject} and averaged across reviewers	x		x
Manuscript type	See *SI Appendix,* Section 3.B and Table S4			x
Key words		x	x	

LS, Life Sciences.

## Results

### Novelty and Acceptance.

We estimate the associations between novelty, conventionality, and acceptance using simple linear probability models* with the following general specification:

[1] $$y_{ij}=\beta_{0}+\beta_{1}n\mathrm{ovelt}y_{ij}+\beta_{2}c\mathrm{onventionalit}y_{ij}+\mathrm{journa}l_{j}+\beta X_{ij}+\varepsilon_{ij},$$

where *y_ij_* = {0, 1} is the outcome for paper *i* submitted to journal *j*, with 1 denoting success (e.g., acceptance or positive review), and the key variables *novelty_i_* and *conventionality_i_* are measured either as percentiles (continuous terms, as shown above) or quintiles (indicator variables, to allow for nonlinearity). The *journal_j_* indicator is a fixed effect for the journal to which the manuscript was submitted and captures stable journal-specific factors (e.g. topics, selectivity) that affect acceptance. To better isolate novelty from confounding factors, we include in the specification a number of manuscript- and author-level covariates captured by the vector ***X_ij_***. The focal and control variables are described in Table 1, which also notes which covariates were available for which of the three datasets—*Cell*, *Cell Reports*, and *IOP*. We estimate these regressions for the pooled sample and each of the three datasets separately using only the controls available in all datasets in the pooled regression and all dataset-specific controls in the dataset-specific regressions. Our preferred specification divides *novelty_i_* and *conventionality_i_* into quintiles to identify possible nonlinearities, which previous research has found (27).

**Fig. 1.**
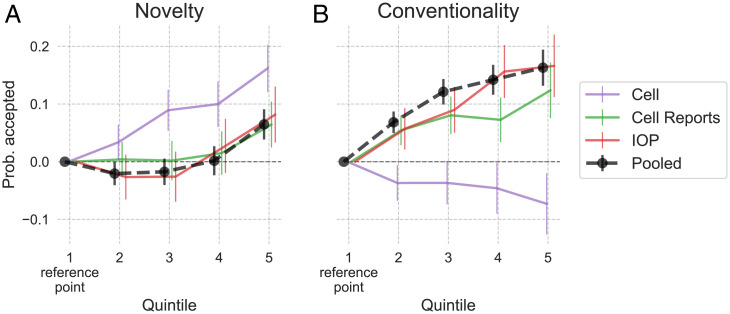
Coefficients from OLS predicting acceptance from novelty (*A*) and conventionality (*B*) and controls. The black curves pool the datasets together, while the other curves use a separate regression for each dataset. Vertical bars are heteroscedasticity-robust 95% CIs. The pooled curves are based on a regression specification that includes only the controls available in all three datasets, whereas the dataset-specific curve specifications include all available dataset-specific controls (Table 1).

Fig. 1 displays the association between acceptance, novelty (Fig. 1*A*), and conventionality (Fig. 1*B*) for the pooled data (black curves) and each dataset separately. The coefficients and 95% CIs for each quintile of novelty are relative to the lowest quintile, set at zero. The full regression table is presented in *SI Appendix*, Table S9. For robustness, we consider alternative specifications in *SI Appendix*, Table S10. Additionally, we use the subset of *Cell* submissions for which we have novelty measured at submission (not publication) and use it to predict acceptance (*SI Appendix*, Table S16). Results from these alternative analyses are qualitatively consistent to the one below.

In the pooled dataset, higher novelty is significantly associated with higher acceptance, and likewise for conventionality. The differences in acceptance are substantial: relative to manuscripts in the lowest quintile of novelty, those in the top quintile are accepted 5.9 percentage points more (CI = [0.033, 0.086], *P* < 0.001). The average acceptance rate in the pooled data is 32%, so 5.9 represents a substantial premium above this baseline. The differences are even more pronounced for conventionality, where relative to the least conventional quintile, the most conventional quintile is accepted 19.9 percentage points more (CI = [0.168, 0.230], *P* < 0.001).

Each separate dataset shows these broad associations, except for *Cell* and conventionality, where higher conventionality is associated with lower acceptance.

In addition to the focus on novelty and conventionality, we consider the number of unique journals a manuscript references, which may reflect its topical breadth and interdisciplinarity. The pooled data and the life sciences journals display a negative association between this variable and acceptance. In the pooled data, a one-SD increase in breadth is associated with a 4.6% decrease in acceptance (*P* < 0.001). That association is 0.1% (*n.s.*) at *Cell,* 4.4% at *Cell Reports* (*P* < 0.001) and +0.14% at *IOP* (*n.s.*) (*SI Appendix*, Table S9, models 2 to 4). This negative albeit nonuniversal association between the number of unique journals referenced and acceptance may be related to previous studies finding poorer evaluations of interdisciplinary research (56, 57).

### Explaining Acceptance of Novelty.

To better understand why novelty is broadly selected for, we disaggregate the review process into three stages: desk review, external peer review (if not desk rejected), and final review. For each stage, we estimate separate ordinary least squares (OLS) regressions for the pooled data and each of the three datasets. The *Cell Reports* dataset did not include external reviewer recommendations and, consequently, it is included only in the desk review panel and excluded from the peer review and final review panels (regressions for the final review panel control for peer reviewer enthusiasm).

**Fig. 2.**
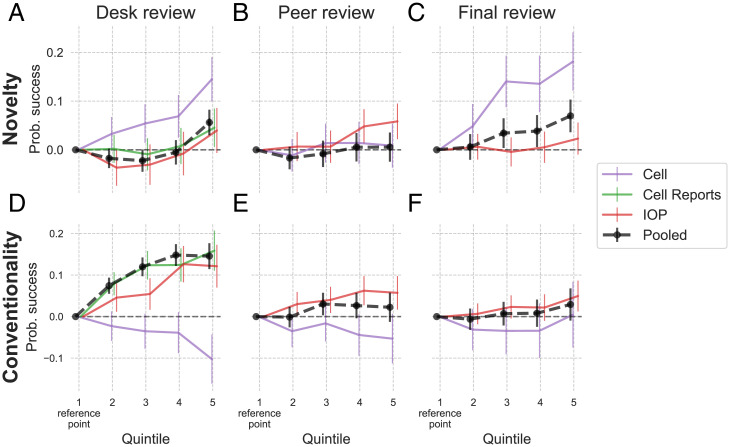
Associations between novelty and success in the pooled sample (black curves), *Cell* (orange curves), *Cell Reports* (green curves), and *IOP* journals (red curves) across the stages of desk review (*A* and *D*), peer review (*B* and *E*), and final review (*C* and *F*). Shown are estimated coefficients and 95% CIs of the four quintile indicator variables. *A–C* show estimates for novelty, and *D–F* show estimates for conventionality. SEs are heteroscedasticity-robust in *A*, *C*, *D*, and *F* and clustered on submission in *B* and *E*. Novelty quintile 1 is the reference point and is set at zero for each journal. The pooled curves are based on a regression specification that includes only the controls available in all three datasets, whereas the dataset-specific curve specifications include all available dataset-specific controls (Table 1).

Estimates from these regressions are displayed in Fig. 2, with the regression table presented in *SI Appendix*, Table S11 and alternative specifications shown in *SI Appendix*, Table S12.

#### Desk review.

Fig. 2 *A* and *D* shows estimates from regressions where the outcome variable is an indicator for whether the paper was sent out for review (i.e., not desk rejected) (*SI Appendix*, Table S11, models 1, 4, 7, and 8). The pooled sample shows that relative to the bottom quintile of novelty, papers in the top quintile are sent out for review 5.6% more often (CI = [0.030, 0.082], *P* < 0.001). The positive association is strongest at *Cell*, where top-quintile papers are sent out for review 14.5% more often. The results are directionally consistent across datasets, although in the physics sample, the top-quintile estimate of 4% is not statistically significant (CI = [−0.006, 0.086], P = 0.092).

In the pooled sample, papers in the top quintile of conventionality are sent out 14.6% more often (CI = [0.114, 0.177], *P* < 0.001). However, the association is negative at *Cell*, where the top-quintile conventionality papers are sent out 10.2% less than the bottom-quintile ones.

Overall, papers highest in novelty and conventionality are most likely to be sent out for review, although the conventionality pattern is heterogeneous across journals.

#### Peer review.

For each reviewer’s recommendation in the initial review round, we define an indicator *positive.review* that takes the values {0 = *reject*, 1 = *accept*, *minor revisions*, or *major revisions*}. In the pooled data, 63.6% of reviews were coded as one. Fig. 2 *B* and *E* shows estimates from regressions where the outcome variable is *positive.review* and SEs are clustered at the submission level (*SI Appendix*, Table S11, models 2, 5, and 9).

In the pooled dataset, reviewers’ recommendations are not strongly associated with novelty or conventionality. However, among the *IOP* journals, top-quintile novelty papers are 5.8% more likely than bottomquintile ones to receive a positive recommendation (CI = [0.022, 0.095], *P* < 0.01). In the same journals, top–conventionality quintile papers are 5.7% more likely than bottom-quintile ones to receive a positive recommendation (CI = [0.017, 0.098], *P* < 0.01). We thus conclude that the relationship between novelty, conventionality, and peer reviewers’ recommendations is weaker than that among editors’ desk decisions, although there is some evidence that the two types of decisions are directionally consistent, favoring papers high in novelty and conventionality.

#### Final review.

Fig. 2 *C* and *F* shows estimates from regressions where the outcome variable is an indicator for whether the paper was accepted and the models are estimated on the subset of submissions that were sent out for peer review (*SI Appendix*, Table S11, models 3, 6, and 10). The specifications include the variable *reviewer.enthusiasm*, the mean of *positive.review* indicators, in order to control for paper quality. There is no curve for *Cell Reports* because our dataset lacked reviewer recommendations.

The patterns show that at this final stage, journal editors select more novel papers, even conditional on peer reviewer enthusiasm. In the pooled dataset, editors accept top–quintile novelty papers 7.0% more than bottom-quintile ones (CI = [0.036, 0.103], *P* < 0.001). The pattern is most pronounced at *Cell*, where the top–quintile novelty advantage is 18.1% (*P* < 0.001).

Conventionality is less strongly associated with editors’ final decisions. In the *IOP* journals, top–quintile conventionality papers are accepted 5% more than bottom-quintile ones (CI = [0.013, 0.087], *P* < 0.01).

A high number of unique journals referenced is generally associated with lower success across the evaluation pipeline. The magnitude of the associations and their statistical significance varies (−0.084, *P* < 0.001 at *Cell* desk review to +0.015, *P* < 0.05 at *IOP* final review), but with the exception of the final review at *IOP*, all coefficients are negative.

Next, we consider several mechanisms discussed in the literature on how novelty and acceptance might be related. These mechanisms include 1) novelty–quality trade-off, 2) novelty and disagreement, and 3) cognitive and strategic biases, and all posit that novelty is selected against. Our data challenge these mechanisms and instead, point to the straightforward mechanism 4) novelty and impact, which posits that novelty is selected for.

#### Trade-offs between novelty, reviewer recommendations, and citations.

Novelty and quality are distinct concepts, where the former is usually considered one of several dimensions of the latter (32, 58, 59). A potential explanation of why peer review might disfavor novelty is if it selects on quality, and novel work is of lower quality on average (24, 51). In other words, there might be a novelty–quality trade-off. We assess this argument in two ways.

First, we consider reviewer recommendations as a proxy of quality. Fig. 2 shows that peer review recommendations are not strongly associated with novelty or conventionality, and to the extent that they are (physics journals), reviewers favor papers that are novel and conventional.

Second, we consider citation impact as a proxy of quality. *SI Appendix*, Table S13 shows estimates from negative binomial models predicting citations after 1 y (models 1 to 3) and 5 y (models 4 to 6). Five-year citations are available for a much smaller set of submissions and only from the *Cell* and* Cell Reports* datasets. However, the more available short-term citations may understate the ultimate impact of novelty, which may have delayed recognition (18).

Novelty is positively associated with 1-y citations (β = 0.00243 to 0.00260 depending on specification, *P* < 0.001). Conventionality is negatively associated with 1-y citations (β = −0.00261 to −0.00300 depending on specification, *P* < 0.001), but the direction of the association is sensitive to the inclusion of controls. In the smaller set of submissions with 5-y citations, novelty and conventionality are both negatively associated with citations, with both associations sensitive to controls. Overall, the data do not provide robust evidence of lower citation impact of novel work.

Taken together, the reviewer recommendation and citation impact data show that more novel submissions are unlikely to be of lower average quality. The estimated citation premium is modest in the 1-y time window but may be considerable over a longer period, as previous research suggests (18).

#### Disagreement and requirement of reviewer consensus.

**Fig. 3.**
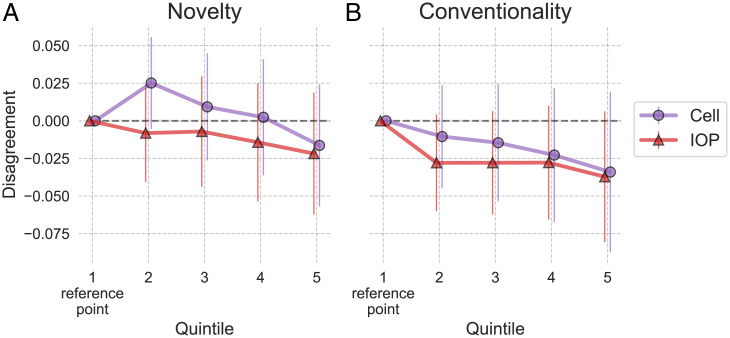
OLS regression coefficients of novelty (*A*) and conventionality (*B*) predicting the amount of reviewer disagreement (SD of binary recommendations in the first review round). “LS” refers to the Life Sciences journals. Standard errors are heteroscedasticity-robust.

Another mechanism hypothesized to drive novelty acceptance is that more novel submissions create more disagreement among reviewers, but selective journals nearly require a consensus of positive reviews, leading them to reject novelty (24). We test this mechanism using peer review data from *Cell*, for which the near-requirement assumption is particularly appropriate, and *IOP* journals. For each submission, we define a variable *disagreement* as the SD of *positive.review* indicators across all reviewers in the first round of review. We regress *disagreement* using specification (1). Fig. 3 displays coefficients of novelty and conventionality quintiles from this regression (*SI Appendix*, Table S14 is the regression table, and alternative specifications are shown in *SI Appendix*, Table S15).

The figure shows that the reviewer disagreement is statistically similar across all novelty and conventionality quintiles. Thus, novelty acceptance is unlikely to be driven by differential disagreement and a near-requirement of consensus.

#### Cognitive and strategic biases.

The cognitive and strategic bias mechanism posits that higher-novelty submissions receive more negative evaluations. Fig. 2 provides evidence against this mechanism. First, Fig. 2*B* shows that reviewers do not substantially disfavor papers of higher novelty. Second, a cognitive bias, if present, should affect both editors and reviewers, who are typically practicing or former researchers. Instead, editors, particularly of *Cell*, show a substantial preference for novelty. These data suggest that cognitive bias against novelty is unlikely to play a major role in these selections. Similarly, the strategic bias mechanisms posit that reviewers, but not editors, should disfavor novel submissions, which may pose a threat to their own ideas. While Fig. 2 does suggest that editors favor novel ideas more than reviewers, it does not support the core implication that reviewers disfavor novel ideas.

## Conclusion

Max Planck, the celebrated physicist and Nobel Prize winner, articulated a common, if pessimistic, view of scientific institutions.

A new scientific truth does not triumph by convincing its opponents and making them see the light, but rather because its opponents eventually die and a new generation grows up that is familiar with it (ref. 60, pp. 33–34).

Contemporary research on scientific funding competitions provides some support for Planck’s view, showing that they tend to select against novelty (16, 24–27). Yet, if novelty is widely disincentivized, why do researchers pursue novel work at all? Previous work suggested that the incentive may be provided by higher long-term citation impact (18, 28, 29) and prizes (5). This paper provides an additional answer—more novel papers are easier to publish. Across 49 journals in the life and physical sciences, including internationally leading titles and those of lower tiers, more novel papers were more likely to be accepted. This preference appeared even among works of similar quality, as reflected by reviewers’ recommendations. Selection on novelty was not unqualified. Papers that lacked conventional elements were less likely to get accepted, so papers that combined novel and conventional elements generally had the best outcomes across the evaluation pipeline.

The fine-grained data enabled direct testing of some of the mechanisms hypothesized to cause conservatism (24). First, we found little-to-no evidence of a novelty–quality trade-off, which would have presented editors and reviewers with a difficult choice. Novel work did not receive worse reviewer recommendations and was cited more in the short term, and while our ability to study long-term citations was limited, existing research shows that it is typically associated with superior long-term impact as well. Second, we found no evidence that novel work generates more disagreement, which could lead to lower acceptance by consensus-seeking editors. Lastly, we found no evidence that cognitive biases leads reviewers and editors to evaluate novel work more negatively. On the contrary, peer reviewers showed no systematic preference against novelty, and those at some journals preferred it.

These findings point to the straightforward mechanism that novel papers generate more impact and are preferred by impact-seeking evaluators (46). The findings also show the importance of editor discretion (i.e., novelty preferences among papers of similar quality). Previous work found that editors exercise discretion in several steps of the evaluation pipeline, including at the final stage after receiving peer reviews (45, 48). Our work shows that some of this discretion takes the form of accepting more novel papers. For instance, editors at *Cell* favored novel papers by 18.1% even conditional on peer reviewer enthusiasm. For selective journals, such a large novelty premium represents a doubling or more of the acceptance rate. Whether this acceptance premium is accounted for by a premium in long-term impact is unclear from our relatively recent data. The findings help resolve a tension inherent in the literature advancing the view that peer review is biased against novelty. If novel works achieve higher citation impact and if the population of citers is similar to the population of reviewers, then to select on novelty in citing but against it in review would imply that these individuals make selections using inconsistent criteria. Yet in our sample of journals the criteria appear consistent.

Lastly, we find that the number of different journals a paper references (normalized by the total number of references) is consistently disfavored by editors and reviewers. This pattern may be related to work on grant review evaluations showing a disfavoring of interdisciplinary projects (56, 57), although future work should investigate this possibility more directly.

This paper is not without limitations that we hope can be addressed in future work. Two of these limitations concern data availability. First, our data did not include peer reviews from *Cell Reports* reviewers. These data would have strengthened (or weakened) our concussions about editor discretion, novelty, and disagreement. Second, we relied primarily on novelty and conventionality that were calculated from references in the published versions of the submitted papers, not the submitted versions. Because many rejected papers could not be linked to their ultimately published versions, we could not calculate novelty for them and excluded them from most analyses. In a subset of papers submitted to *Cell* for which we had the originally submitted versions, the correlation between the submitted and published measures was high (+0.75 for both novelty and conventionality). Furthermore, in that subset, novelty at submission was predictive of acceptance (*SI Appendix*, Table S16). We believe these results make it unlikely that our results are severely biased by missing data on rejected papers.

Second, the study uses quantitative measures of complex constructs, like novelty and quality, and our understanding of these measures is evolving (61–63). Further specification and validation of the measures are undoubtedly needed. However, it should be noted that relying on qualitative judgments comes with its own difficulties, since these judgments may be biased by novelty or other factors.

Third, more research is needed to understand heterogeneity in journals’ responses to novelty and conventionality, such as the preference of *Cell* editors against conventionality at the desk review stage. While our data could rule out some mechanisms, more is needed to rule mechanisms in. For example, editors of top journals may favor novelty and disfavor conventionality to differentiate the journals from competitors or because their audiences are more tolerant of both (64). On the other hand, given the literature on the cognitive burden of evaluating novel ideas, it is possible that *Cell* represents a relatively rare case in which evaluators are properly resourced (*e.g*., have enough time and expertise to evaluate complex and novel ideas fully). Such contextual research is needed to advance theory and make our work more directly applicable to specific organizations. Lastly, although this work uses a substantial sample of journals from the life and physical sciences, data from other fields would help increase generalizability.

Given the voluminous literature advancing that view that peer review is biased against novelty, it is important to consider what may account for our disparate conclusion. First, it is possible that previous work was affected by selection biases. For example, case studies of peer review tend to focus on instances where it failed to properly value novel work (33, 35), not cases where it succeeded. Second, previous work may have been affected by confounding. If novel ideas have lower average quality at earlier stages of the research pipeline, such as in grant competitions (51), and analysts do not fully account for differences in quality, then selections against low quality work may be misinterpreted as selections against novelty. Third, cognitive biases may be stronger at earlier stages, where uncertainty is highest.

Lastly, a model by Gross and Bergstrom (44) provides a compelling account of why our results depart from the bulk of the literature. The model shows why even if the scientific community generally favors novel ideas that surprise and change prior beliefs and evaluators are unbiased, they may select against novelty in *ex ante* review (i.e., grant competitions) but for it in *ex post* review (i.e., manuscript review). For an idea to change evaluators’ beliefs, those beliefs must be predisposed against it initially. In *ex ante* review, creators of the idea lack a mechanism by which to persuade appropriately skeptical reviewers. In *ex post* review, they have that persuasion mechanism—the results of the research.

In sum, our results illuminate conservatism in science, showing that it is unlikely to be caused by institutions using a selection method — peer review — that is inherently biased against novelty. The exact format in which peer review is deployed, and whether in early or late stages, may still contribute to conservatism, but it is important for future research to also consider other drivers.

## Data and Methods

We use three datasets provided by Cell Press and Institute of Physics Publishing (IOP).

### *Cell Press*: Life Sciences Journals.

The Elsevier (*Cell Press*) datasets cover 17,504 submissions to *Cell* and 13,905 submissions to *Cell Reports* between 2013 and 2018. These data are supplemented with bibliometric data from the Microsoft Academic Graph (MAG), accessed in June 2019. MAG is among the largest and most widely used bibliometric databases (65, 66). *Cell*’s submissions data are more complete than *Cell Reports*', and include in most instances the peer review recommendations and, in some instances, the original submission files. There are substantial missing data particularly on rejected submissions for both journals (discussed below), resulting in a smaller dataset actually used in the analysis, which we call the “analytic sample.” Additionally, we excluded from the analytic sample 1) 180 submissions known to be commissioned by the editors, as these likely had an atypical evaluation process (i.e., unlikely to be desk rejected), and 2) 414 submissions terminated during the evaluation or publication process. The analytic sample consists of 8,435 submissions to *Cell* and 12,103 submissions to *Cell Reports*. A comparison of the full and analytic samples on key covariates is presented in *SI Appendix* (*SI Appendix*, Tables S2 and S3).

Beyond similar scope, the journals use nearly identical review processes. Both are single anonymous (i.e., authors’ identities are visible to the editors and reviewers). Editors of both journals first evaluate submissions at the “desk” before deciding whether to send them out to peer reviewers and desk reject a substantial number of submissions. Reviewer instructions are similar, and both journals use a standard commercial editorial software. Interviews with editors indicate that papers are reviewed by similar numbers and types of reviewers, with the caveat that *Cell* is likely able to recruit more prominent reviewers if needed. The journals have no editors in common.

#### *Cell*.

The data include all submissions between 2013 and 2018, including the document object identifier (DOI) for manuscripts that were ultimately published in *Cell* or published elsewhere and successfully located by a third-party contractor. The contractor used the submission title and author information to locate submissions rejected and published elsewhere. Of the 17,509 submissions, 10,454 (59.7%) were successfully matched to a DOI. The unmatched submissions were primarily those rejected by *Cell*, of which 46.1% were unmatched, whereas of the accepted submissions, only 4.9% were unmatched. Overall, the analytic sample is missing about half of the rejected submissions.

The DOI was then used to collect additional data on the submissions from MAG. These additional data include author productivity, paper topics, paper citations, and novelty.

*Cell* data include peer review recommendations for manuscripts that were sent out for external review. A manuscript can be reviewed by several referees and over several rounds of review. In our analyses, we focus on the first review rounds only.

The data also include a number of original submission files, including the PDF or Word file of the main manuscript. Although we rely on the published versions of submissions in the analysis, we use this smaller sample to estimate the degree to which the published versions depart from the submitted ones (*Submitted vs. published versions*).

#### *Cell Reports*.

The data for *Cell Reports* were less complete. They were missing peer reviewer recommendations for all submissions and the year of submission for some submissions. They also include only submissions successfully matched to a DOI, whether published in *Cell Reports* or rejected and published elsewhere, as matched by the same third-party contractor. No information was available for rejected but unmatched papers. Thus, whereas in the *Cell* data we can estimate the amount of missing data due to unmatched submissions, we cannot do so with *Cell Reports*. We expect that similarly to *Cell*, a substantial fraction of *Cell Reports* rejected submissions was unmatched and excluded from our analytic sample.

We impute the missing years of submission for accepted papers by using the years of publication (available for all submissions) and subtracting the median time to publication estimated from the more complete data for *Cell* (median equals 1 y).

#### Submitted vs. published versions.

Many of the key variables in our analysis come from MAG, which contains data primarily on published papers. In particular, to calculate novelty and conventionality, we use reference lists from the papers’ published versions. A limitation of using papers' published rather than submitted versions is that some of their characteristics, like novelty, may change during the review process. There are several reasons to believe that this limitation does not affect our analysis substantively. First, a number of studies across a number of fields find that the core content of manuscripts tends to change little from submission to publication, with most changes occurring in framing (67, 68), tone (69, 70), and formatting (71). Second, some important manuscript characteristics, such as the key words and number of authors, are unlikely to change substantially from submission to publication. Lastly, we use the subset of 2,778 *Cell* submissions for which we have both submitted and published manuscript versions to track how our key manuscript characteristic—novelty—changed. The analysis presented in *SI Appendix*, *Submitted vs. Published Versions* shows that the correlation between submitted and published novelty, measured in percentiles, is +0.75. Conventionality shows an equally strong correlation. Additionally, we use the subset with novelty at submission to predict acceptance, finding the results consistent with novelty at publication (*SI Appendix*, Table S16). These similarities support relying on published novelty as a reasonable but imperfect proxy for submitted novelty.

### *IOP*: Physical Sciences Journals.

Review data on physics journals were provided by the *IOP*, one of the world’s largest publishers, which one analysis found to account for nearly 10% of papers indexed in the Web of Science in 2013 (72). These data included peer reviews but did not include initial submitted versions of the papers or key words. While the full dataset covered 44,350 papers submitted in 2018, most of these were not located in our 2019 version of MAG (never published, published but not indexed, etc.) or were indexed but missing data on references (*SI Appendix*, *Full vs. Analytic Samples* has details). The analytic sample used in the analyses consisted of 6,785 papers. *SI Appendix*, Table S8 shows the number of submissions in the analytic sample by journal. The journals varied in 2018 impact factor, ranging from *2D Materials* with an impact factor of 7.343 to *European Journal of Physics* with an impact factor of 0.861, and a few newer journals did not yet have an impact factor.

The *IOP* submission data provide the DOIs for all accepted papers, which we located in MAG if they were indexed. For rejected submissions, we searched the title of the paper in the OpenAlex database (73) to identify the DOI and then, used the DOI to locate each paper in the MAG to obtain additional data as before. We used the same pipeline to calculate novelties as in the life sciences datasets.

### Measures.

#### Novelty and conventionality.

We use the measure proposed by Uzzi et al. (29), which focuses on atypical combinations (ACs) of journals referenced in an article. We use this measure due to its wide use and concordance with qualitative evaluations of research novelty (61). Additional discussion of validation is in *SI Appendix*, *Novelty Measure*.

AC is defined at the journal-pair-year level; AC for two journals in a given year is measured by comparing its observed cocitation frequency across all papers published that year with those expected from the following null model. In the null model, journal–journal citations are rewired at random, with the constraint that the number of outgoing and incoming citations remains constant (i.e., journals that are cited often remain cited often in the random network). Specifically,

$$AC_{}(\mathrm{journa}l_{i},\;\mathrm{journa}l_{j},\;\mathrm{yea}r_{k})=[\mathrm{observe}d_{\mathrm{ijk}}-\mathrm{mean}(\mathrm{expecte}d_{\mathrm{ijk}})]/SD(\mathrm{expecte}d_{\mathrm{ijk}}),$$

where *journal_i_* and *journal_j_* are any two journals from a submission’s reference list; *k* is the year of submission; *observed_ijk_* is the number of times the two journals were cocited by all papers published in the literature in year *k*; and *expected_ijk_* is the number of cocitations expected from a random citing process, which is repeated 10 times. From the 10 runs, AC calculates the mean and SD. Lower *AC_ijk_* occurs when two journals are expected to be cocited often and therefore, have high *mean*(*expected_ijk_*), but they are in practice cocited infrequently and therefore, have low *observed_ijk_*. This case occurs when two journals are both prominent and often cited, but they are far from each other in conceptual space and are infrequently cocited. Intuitively, we expect that cociting such journals corresponds to an atypical, or novel, conceptual intervention.

This method gives each submission a distribution of ACs, one for each journal pair in its reference list, and year *k* set to the submission year. We follow the Uzzi et al. (29) paper in using two summary statistics of this distribution: 1) the median (conventionality) and 2) 10th percentile (novelty). We reverse code novelty so that higher values correspond to more ACs, while higher conventionality values correspond to more typical combinations. These two summary statistics are highly correlated (*SI Appendix*, Table S7), and we depart from ref. 29 by excluding in our analyses the interaction term between novelty and conventionality due to our more limited sample size that prevents robust estimation of interactions. Lastly, we convert novelty and conventionality summary statistics into percentiles (separately across life sciences and physics datasets to account for broad domain differences). In the end, each paper is characterized by one novelty percentile and one conventionality percentile.

#### Proxies for manuscript quality.

To distinguish novelty and quality, we rely on three proxies for quality. First, we use authors’ reputations, measured as the number of publications in total and in the journals to which they have submitted. These data were missing for the majority of *IOP* submissions. Consequently, we use author reputation data only in the *Cell* and *Cell* *Reports* analyses. Second, we use peer reviewer enthusiasm measured via reviewers’ recommendations, with one equal to accept or revise and resubmit and zero equal to reject. Third, we use 1- and 5-y citations.

#### Number of unique journals referenced.

To distinguish novelty, which is based on combinations of journals, from the quantity of those journals, we measure the number of unique journals as manuscript references. We normalize this number by the number of references overall to account for different reference list lengths between manuscript types or topical areas. Controlling for this measure addresses the concern that a high number of unique journals referenced in a submission may generate atypical journal combinations purely mechanically. Indeed, the correlation between novelty and the number of unique journals in our data is +0.50. Using both measures thus helps isolate the effects of novelty conceptually and statistically.

#### Topics.

Topic choice may affect acceptance of scientific works (i.e., manuscripts on trending topics may be more likely to be accepted). We sought to control for research topics using keywords, which we were able to do in the *Cell Press* datasets only. Key words were especially important for the *Cell Press* journals due to their broad scope. (For the *IOP* journals, many of which had a narrower scope, we rely on journal fixed effects.) MAG provides a list of key words for each paper with associated confidence scores between zero and one. For each journal, we focused on the most common key words, specifically those used by at least 500 submitted papers (accepted and rejected). There are 13 and 26 such key words for *Cell* and *Cell Reports*, respectively. Each key word was used as a continuous variable in the regression, taking on the value of the key word's confidence score for that paper.

#### Last author characteristics.

The journals in all three datasets use single-blind reviewing. Consequently, author information may influence editors’ and reviewers’ perception of manuscript quality directly (74, 75) or indirectly via possible associations with novelty (76, 77) and topics (78). To better isolate the association between novelty and acceptance from these additional acceptance pathways, we control for the following author-related submission characteristics, which were available for *Cell Press* journals only: last author's number of total previous publications and previous publications at *Cell* or *Cell Reports* up to the submission year (Table 1).

## Supplementary Material

Supplementary File

## Data Availability

The raw data are not publicly available to protect individuals' privacy and due to restrictions imposed by the data use agreements. Anonymized data that may be used to replicate the findings are provided at https://doi.org/10.7302/ack7-as60.

## Footnotes

^*^Logistic regression models yield qualitatively similar results and are provided in *SI Appendix*, Table S15.
