# Supplementary material for: Is novel research worth doing? Evidence from peer review at 49 journals
Source: Proc Natl Acad Sci U S A. 2022 Nov 17;119(47):e2118046119. doi: 10.1073/pnas.2118046119 (PMC9704701; doi:10.1073/pnas.2118046119)
Supplement: Supplementary File [file pnas.2118046119.sapp.pdf]

Supplementary Information for

"Is novel research worth doing? Evidence from peer review at 49 journals"

Table of contents

1. Novelty measure
   A. Replicating Uzzi et al. (2013) on different data
   B. Validation using article types
   C. Additional validation from peer review
2. Submitted vs. published versions of *Cell* manuscripts
3. Full vs. analytic samples
   A. *Cell Press* journals
   B. *IOP* journals
4. Supplementary tables
5. Supplementary references

1. Novelty measure

A. Replicating Uzzi et al. (2013) on different data

We used the Microsoft Academic Graph (MAG), accessed in June 2019, to gather data on submissions' authors, citations, and references, the latter of which are used in the novelty metric. MAG is among the largest bibliometric databases, with much more comprehensive coverage than commonly used commercial databases like Web of Science (WoS) and Scopus (Visser et al., 2021). However, the latter are generally more selective than MAG in the items they index. In contrast, MAG contains a number of non-English works (Visser et al., 2021) and even some patents.

Our goal was to match as closely as possible the novelty ("atypical combinations") calculations of Uzzi et al. (Uzzi et al., 2013), hereafter "UMSJ," using a different bibliometric dataset. UMSJ used all research articles published over the 1950-2000 period by 15,613 journals indexed in WoS. Our version of MAG indexed 48,757 journals which cover journal articles, working papers, book chapters, and other article types. We sought to take a subset of MAG that was similar to WoS but still using freely accessible data. To do so, we used a third database, Scopus, which has been shown by Visser et al. (Visser et al., 2021) to overlap very well with the journals indexed by WoS. Journals indexed by Scopus are publicly available[1]. There were 21,357 MAG journals that are indexed by Scopus, matched on ISSN. Keeping only these journals in the "overlap" dataset, we call it the "MAG-Scopus" subset. In calculating novelty, we consider a paper's references only within this set of ~21K MAG-Scopus journals. The literature pool for the novelty calculation consists of 10,144,021 citing papers published in the 2013-2019 period, and their 311,074,438 cited papers, keeping only those cited papers published after 1950, as in USMJ.

To assess whether USMJ replicates using different data, we replicated their analysis of the relationship between novelty and citation impact. USMJ showed that papers exhibiting both high tail novelty and high median conventionality are twice as likely to be "hits" -- receive citations in the top 5% of that year's citation distribution. We calculated novelties of 5,211,245 papers published between 1990 and 2000 in MAG-Scopus and their 89,486,372 references within that subset, and as in UMSJ grouped papers into four bins according to whether their tail/median novelty was above/below the median. We then measured these papers' citations 8 years later, and recorded the fraction of "hit" papers in each group. To test whether our findings applied to more recent data, we also repeated this exercise for the 1,235,329 MAG-Scopus papers published in 2010. The groups and fractions are displayed in Table S1, alongside corresponding fractions from USMJ.

[1] The list was obtained in 2019. The current list can be accessed via https://www.scopus.com/sources.uri, "Download Scopus Source List". Last accessed 20121-05-17.

	Low tail, high median	Low tail, low median	High tail, high median	High tail, low median
**MAG-Scopus 1990-2000**	2.57	5.51	5.53	7.70
**MAG-Scopus 2010**	2.69	5.56	5.30	7.35
**UMSJ (WoS)**	2.05	5.82	5.33	9.11

Table S1. *Percent of citation hit papers, by novelty group (columns), computed on three different datesets (rows). "Hit" is defined as having the 8-year citation count fall in the top 5% of that year's citation distribution. Low/high tail and median novelty are values below/above the median.*

The MAG-Scopus results match USMJ qualitatively. The ordering of groups by percent of citation hits is the same for MAG-Scopus 2010 and similar for MAG-Scopus 1990-2000, aside from the two middle groups, which are only slightly different from each other in the original USMJ. Across all datasets, the exercise supports USMJ's main claim that papers with high tail novelty and high median conventionality display a hit probability about twice as much as papers that are high on either of the two dimensions. Overall, these results suggest that our novelty measure based on the MAG literature filtered by Scopus journals works similarly to WoS data, and applies to more recent literature.

B. Validation using research articles vs. reviews

As one validation check of whether USMJ measures novelty, we consider its distribution by article types expected to be more or less novel: primary research (*i.e.* research articles) and reviews. We expect reviews, being syntheses of primary research from the recent past, to have lower novelty and higher conventionality than primary research. Data on article type was missing for 7717 submissions across the two Life Sciences journals[2], and these were excluded from this analysis. Among the observations with a valid article type, 21190 were primary research and 120 were reviews. Figure S1 below displays the boxplot of novelty and conventionality percentiles by article type. As predicted, the novelty of primary research was on average 6.9 percentiles higher than that of reviews (Wilcoxon rank-sums test statistic=2.13, p=0.033) and the conventionality was 13.3 percentiles lower (Wilcoxon rank-sums test statistic=-4.27, p<0.001), supporting the interpretation of USMJ measures as novelty and conventionality.

[2] Most of the missing article types were among articles accepted by *Cell Reports*. An examination of 200 randomly chosen papers in this set revealed all 200 to be of type "Article" in the Scopus database, accessed 2021-05-18.

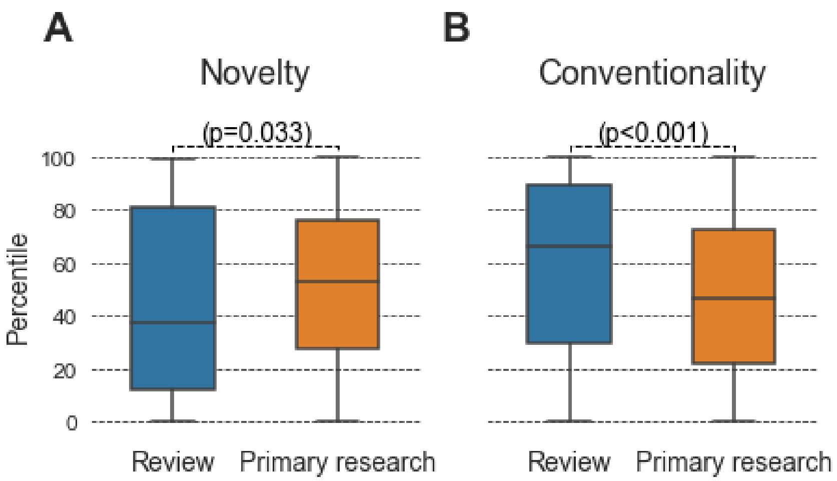

Figure S1. *Boxplot of novelty percentiles (**A**) and conventionality (**B**) by article type in the Life Sciences journals.*

C. Additional validation from peer review

Additional support for the interpretation of USMJ as a proxy of novelty comes from (Bornmann et al., 2019). Bornmann et al. calculated a version of USMJ that is revised by Lee et al. (Lee et al., 2015). The Lee *et al*. revision retains the essence of USMJ, but simplifies the normalization of atypical combinations, *i.e*. does not randomly rewire the year-specific journal-journal co-citation network as USMJ and the present paper do. Bornmann *et al*. then compared these revised-USMJ measures to qualitative rankings from members of Faculty of 1000, a post-publication peer review system. A key result is that a one SD-increase in revised-USMJ novelty corresponds to a 7.47% increase in the number of reviewers labeling the paper as presenting a "new finding." This result suggests a correspondence between USMJ and qualitative perceptions of novelty post-publication.

2. Submitted vs. published versions of *Cell* manuscripts

While it may be desirable to use metrics derived from the submitted versions of manuscripts to study editorial decision-making, our data include the submitted manuscript files (in PDF or Word format) only for submissions to *Cell*, and only for two years. Lacking submitted versions of most of the submissions, we thus relied on the published versions of articles as a proxy for their submitted versions. However, for the subset with both submitted and published manuscript versions, we can compare how novelty metrics changed.

This subset consists of 3,717 articles submitted to *Cell* in 2013-2014. We used the *Grobid* (Introduction - GROBID Documentation, n.d.) package to parse the manuscript files and extracted references from them. *Grobid* uses the *CrossRef* API service to resolve the references in text into DOI. We were able to extract references for 2,778 initial submissions.

Whereas submitted-version novelties may be ideal in theory, in practice, accurately extracting references from PDF and Word files is error-prone. There are three main ways in which novelty calculated from the submitted and published versions may differ:

1. True changes in the references
2. Extraction errors from submitted versions: our pipeline may have failed to successfully identify or parse references in the submitted version. The *Grobid* documentation shows f-scores in the range of 0.75 - 0.89 (Introduction - GROBID Documentation, n.d.).
3. References included in the calculation of novelty from of the published version consist of all those with a valid MAG ID, but not necessarily a DOI. In contrast, references included in the calculation of novelty from the initial version consist of all those with a valid DOI (because they use CrossRef), but not necessarily MAG ID.

We display scatter plots of novelties and conventionalities calculated from the published vs. submitted versions of papers in Figure S2. Panel A shows raw novelty (*z*-scores, calculated as in Uzzi et al. (2013)) of submitted vs. published versions, and Panel B shows percentiles of novelty for published vs. submitted versions. Panels C and D repeat the plots for conventionality.

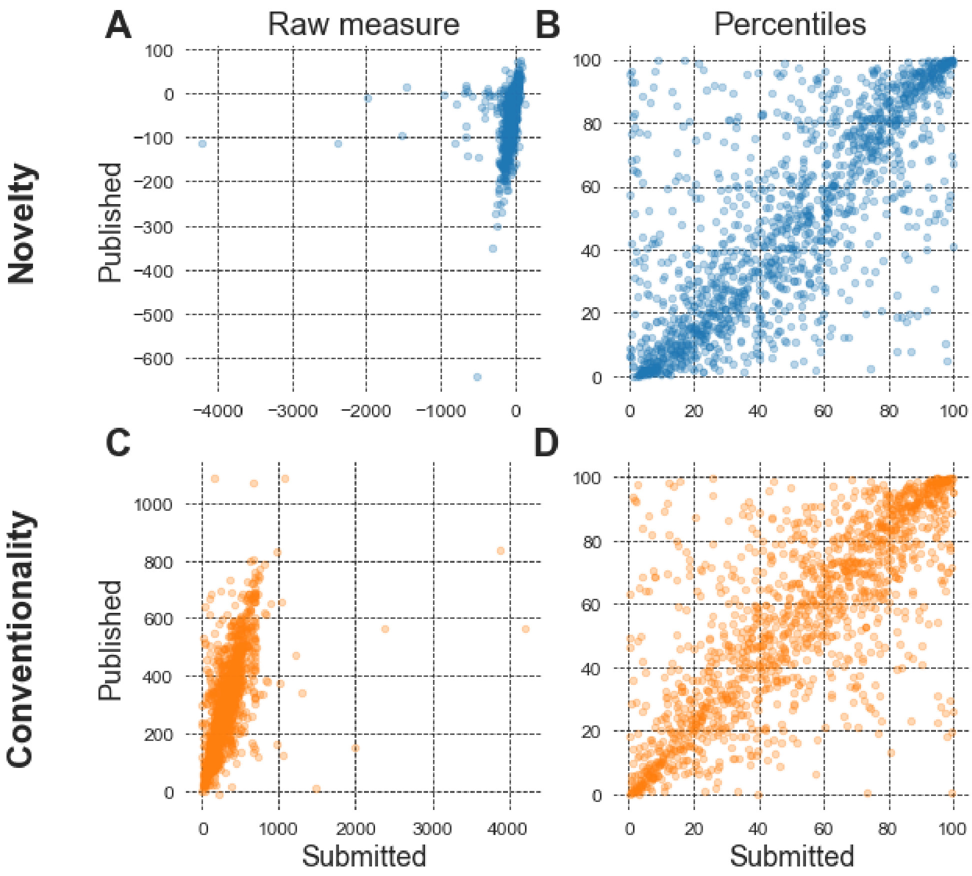

Figure S2. *Scatter plot of novelties (top row) and conventionalities (bottom row), using raw versions calculated as in Uzzi et al. (2013)) (left column). In each panel, the x-axis is the measure calculated from the paper's submitted version, and the y-axis is from the published version.*

The Pearson correlation for raw novelty scores is 0.28 (p<0.001) and the Pearson correlation for percentiles is 0.75 (p<0.001). For conventionality, the corresponding values are 0.59 (p<0.001) and 0.75 (p<0.001).

We interpret the much smaller correlation for raw novelties and conventionalities as revealing the sensitivity of these metrics to small changes in references, whether due to true changes or parsing and database errors, and which is evidenced by several extreme outliers. In contrast, percentiles are robust to outliers, and show that novelty and conventionality are relatively consistent across the publication process. Consequently, we rely on percentiles in the rest of our analysis.

3. Full vs. analytic samples

The primary difference between the full and the (smaller) analytic samples for both the *Cell Press* and *IOP* datasets is that the analytic samples include only submissions for which we were able to calculate novelty. Novelty calculation for a submission were often not possible because it was rejected and a published version was not located (*e.g.* never published) or it was published but not indexed in MAG.

A. *Cell Press* journals

Tables S2 and S3 below show how key covariates vary between the full and analytical samples for *Cell* and *Cell Reports*. The last column of the tables shows the standardized difference, *i.e.* difference in means divided by the pooled standard deviation. The differences are modest, never exceeding a third of the pooled standard deviation. The largest differences are observed in quality, measured by the mean reviewer recommendation (0=reject, 1=accept or revise-and-resubmit), and the number of references, with both being higher in the analytic sample. This pattern is likely explained by lower quality submissions being more likely to be rejected, and subsequently less likely to be matched to MAG, whether due to never being published or published under a significantly different title.

Cell				
	**Full sample mean**	**Analytic sample mean**	**diff.**	**std. difference**
**Count**	17324	8657		
**Mean reviewer recommendation**	0.411	0.482	-0.071	-0.223
**Num. unique journals ref'd (standardized)**	-0.235	-0.247	0.012	0.013
**Number of references**	48.78	55.205	-6.426	-0.264
**Number of *Cell* refs.**	3.118	3.529	-0.411	-0.13
**Citations after 2 years**	13.187	14.279	-1.091	-0.059
**Citations after 5 years**	89.034	90.492	-1.458	-0.012
**Year submitted**	2015.697	2015.434	0.263	0.162
**Number of authors**	10.245	10.159	0.086	0.011
**Number of last authors' pubs.**	117.451	118.712	-1.261	-0.009
**Number of last author's *Cell* pubs**	3.422	3.623	-0.201	-0.028

Table S2. *Covariates and their differences in the full and analytic samples for submissions to* Cell.

Cell Reports				
	**Full sample mean**	**Analytic sample mean**	**diff.**	**std. difference**
**Count**	13905	12103		
**Num. unique journals ref'd (standardized)**	0.6	0.598	0.001	0.011
**Number of references**	41.975	47.694	-5.719	-0.305
**Number of *Cell Reports* refs.**	0.393	0.446	-0.054	-0.07
**Citations after 1 years**	4.604	5.199	-0.595	-0.098
**Citations after 5 years**	28.525	30.072	-1.547	-0.054
**Year submitted**	2015.607	2015.555	0.052	0.035
**Number of authors**	10.323	10.371	-0.049	-0.002
**Number of last authors' pubs.**	101.664	102.002	-0.338	-0.003
**Number of last author's *Cell Reports* pubs**	0.424	0.439	-0.016	-0.015

Table S3. *Covariates and their differences in the full and analytic samples for submissions to Cell Reports.*

B. *IOP* journals

We exclude from the *IOP* analytic sample manuscript types that are likely to undergo an unconventional review process or not present original research, such as Corrigendum (n=580) and Perspective (n=504). The top 5 manuscript types remaining in the *IOP* analytics smaple are shown in table S4.

Type	Count
Paper	38112
Letter	2309
Special Issue Article	2095
Topical Review	1017
Note	382

Table S4. *Manuscript types of submissions to IOP journals in 2018 in the full sample.*

Table S5 presents the difference between the *IOP* analytic and full samples on key covariates. The analytic sample is much smaller than the full sample for two main reasons. First, we attempt to match 2018 submissions, whether accepted or rejected, to the mid-2019 MAG and the majority of these (63.1%) were not indexed or located by that time. It is possible they were published and indexed in later versions of MAG, or will be published in the future. Second, an additional 21.5% of submissions were located in MAG but had no data on references.

The standardized differences (last column) are modest, never exceeding a quarter of the pooled standard deviation. The analytic sample has slightly more positive reviewer recommendations and citation counts, suggesting that it consists of slightly higher quality papers. This selection bias is likely due to higher quality papers being more likely to be accepted for publication, leading to higher probability and speed of indexing in MAG.

IOP Journals				
	**Full sample mean**	**Analytic sample mean**	**diff.**	**std. difference**
Number of submissions	44350	6785		
Number of unique journals	50	47		
Number of authors	4.418	4.915	-0.496	-0.174
Num. unique journals ref'd (std.)	-0.001	0.126	-0.126	-0.13
Number of references	27.502	29.836	-2.335	-0.144
Reviewer recommendation (binary) mean	0.699	0.794	-0.095	-0.248
Citations after 1 years	0.558	0.734	-0.176	-0.122
Year submitted	2018	2018		

Table S5. *Covariates and their differences (raw and standardized) in the full and analytic samples for submissions to IOP journals.*

4. Supplementary tables

Table S6. *Descriptive statistics for the analytic sample of the pooled data.*

	count	mean	std	min	25%	50%	75%	max
**Novelty percentile**	27,545.00	50.10	28.82	0.01	25.18	50.14	75.07	100.00
**Conventionality percentile**	27,545.00	49.86	28.85	0.00	24.86	49.82	74.84	100.00
**Num. unique journals ref'd (std.)**	27,545.00	0.03	0.93	-3.24	-0.59	0.02	0.65	3.32
**Num. of references**	27,545.00	45.66	19.60	2.00	33.00	45.00	57.00	316.00
**Num. of authors**	27,545.00	8.96	15.35	1.00	5.00	7.00	11.00	759.00
**Year submitted**	27,545.00	2,016.12	1.66	2,013.00	2,015.00	2,016.00	2,018.00	2,018.00
**Citations after 1 year**	26,366.00	6.88	12.39	0.00	1.00	3.00	8.00	333.00

Table S7. *Correlation table for the analytic sample of the pooled data.*

	**Novelty percentile**	**Conventionality percentile**	**Num. unique journals ref'd (std.)**	**Num. of references**	**Num. of authors**	**Year submitted**	**Citations after 1 year**
**Novelty percentile**	1.00	-0.81	0.52	0.01	0.07	0.02	0.01
**Conventionality percentile**	-0.81	1.00	-0.67	0.03	-0.06	-0.06	0.04
**Num. unique journals ref'd (std.)**	0.52	-0.67	1.00	-0.41	0.02	0.07	-0.16
**Num. of references**	0.01	0.03	-0.41	1.00	0.09	-0.24	0.26
**Num. of authors**	0.07	-0.06	0.02	0.09	1.00	-0.07	0.11
**Year submitted**	0.02	-0.06	0.07	-0.24	-0.07	1.00	-0.30
**Citations after 1 year**	0.01	0.04	-0.16	0.26	0.11	-0.30	1.00

Table S8. *IOP journals ("Physics dataset"), 2018 journal impact factors, and the corresponding number of submissions in the full and analytic samples.*

	Impact factor	Full #	Analytic #
**Materials Research Express**	1.449	7092	1034
**Nanotechnology**	3.399	3897	1046
**Journal of Physics D: Applied Physics**	2.829	3734	614
**Journal of Physics: Condensed Matter**	2.711	2475	293
**Journal of Physics A: Mathematical and Theoretical**	2.11	1804	196
**New Journal of Physics**	3.773	1728	264
**Smart Materials and Structures**	3.543	1666	378
**Measurement Science and Technology**	1.861	1646	267
**Physics in Medicine & Biology**	3.609	1547	302
**Physica Scripta**	2.151	1283	93
**2D Materials**	7.343	1181	227
**Classical and Quantum Gravity**	3.487	1044	175
**Journal of Optics**	2.753	1011	177
**Semiconductor Science and Technology**	2.654	975	142
**European Journal of Physics**	0.861	829	78
**Journal of Physics B: Atomic, Molecular and Optical Physics**	2.115	747	77
**Plasma Sources Science and Technology**	3.584	747	90
**Nonlinearity**	1.729	732	80
**Modelling and Simulation in Materials Science and Engineering**	1.826	665	82
**Journal of Micromechanics and Microengineering**	2.141	593	112
**Physiological Measurement**	2.246	574	57
**Journal of Neural Engineering**	4.551	554	124
**Superconductor Science and Technology**	3.219	536	82
**Biofabrication**	7.236	533	121
**Biomedical Physics & Engineering Express**	n/a	496	61
**Biomedical Materials**	3.44	470	102
**Plasma Physics and Controlled Fusion**	2.799	444	47
**Journal of Physics Communications**	n/a	435	38
**Inverse Problems**	1.858	425	77
**Journal of Physics G: Nuclear and Particle Physics**	3.534	414	38
**Physics Education**	n/a	367	40
**Bioinspiration & Biomimetics**	3.13	344	60
**Journal of Radiological Protection**	1.327	321	39
**Quantum Science and Technology**	3.022	206	24
**Journal of Breath Research**	3	161	33
**Methods and Applications in Fluorescence**	2.94	156	31

**Surface Topography: Metrology and Properties**	2.439	146	4
**Physical Biology**	1.818	131	14
**Journal of Physics: Materials**	n/a	97	7
**Nano Futures**	3.306	80	19
**Plasma Research Express**	n/a	78	8
**Flexible and Printed Electronics**	3.588	77	11
**Environmental Research Communications**	2.104	58	3
**Journal of Physics: Energy**	5.967	54	6
**Multifunctional Materials**	n/a	50	4
**Electronic Structure**	n/a	38	2
**Journal of Physics: Photonics**	n/a	35	6

Table S9. *OLS regression models predicting acceptance in 1) the pooled dataset, 2)* Cell*, 3)* Cell Reports*, and 4)* IOP *journals. Standard errors are heteroscedasticity-robust. The models vary in the controls they use, which are noted at the bottom of the table.*

	Dependent variable:is_accepted			
	Pooled	Cell	Cell Reports	IOP
	(1)	(2)	(3)	(4)
novelty 2nd quintile	-0.021*	0.034*	0.004	-0.027
	(0.010)	(0.015)	(0.015)	(0.020)
novelty 3rd quintile	-0.018	0.089***	0.002	-0.026
	(0.012)	(0.018)	(0.017)	(0.022)
novelty 4th quintile	0.002	0.100***	0.015	0.027
	(0.013)	(0.020)	(0.019)	(0.024)
novelty 5th quintile	0.065***	0.162***	0.064**	0.082***
	(0.013)	(0.021)	(0.020)	(0.025)
conventionality 2nd quintile	0.068***	-0.037*	0.054***	0.057**
	(0.010)	(0.016)	(0.013)	(0.018)
conventionality 3rd quintile	0.121***	-0.037	0.081***	0.090***
	(0.011)	(0.019)	(0.017)	(0.020)
conventionality 4th quintile	0.142***	-0.046*	0.073***	0.156***
	(0.013)	(0.022)	(0.020)	(0.023)
conventionality 5th quintile	0.163***	-0.073**	0.124***	0.166***

	(0.016)	(0.027)	(0.025)	(0.028)
Num. authors	0.002***	0.002*	0.001***	0.024***
	(0.000)	(0.001)	(0.000)	(0.002)
Num. unique journals ref'd (std.)	-0.046***	-0.071***	-0.044***	-0.004
	(0.005)	(0.008)	(0.007)	(0.010)
Num. references	-0.002***	-0.000	-0.004***	-0.007***
	(0.000)	(0.000)	(0.000)	(0.000)
Journal	Yes	No	No	Yes
Year submitted	Yes	Yes	Yes	No
Keywords	No	Yes	Yes	No
Last author pubs	No	Yes	Yes	No
Last author pubs in journal	No	Yes	Yes	No
Manuscript type	No	No	No	Yes
Num. refs to journal	No	Yes	Yes	No
Observations	27,545	8,657	12,103	6,785
R^2	0.116	0.179	0.259	0.197
Adjusted R^2	0.114	0.176	0.256	0.190
Residual Std. Error	0.462 (df=27480)	0.396 (df=8624)	0.429 (df=12057)	0.450 (df=6727)
F Statistic	89.216*** (df=64; 27480)	67.149*** (df=32; 8624)	194.687*** (df=45; 12057)	28.927*** (df=57; 6727)
Note:				*p<0.05; **p<0.01; ***p<0.001

Table S10. *OLS regression models predicting acceptance in the pooled dataset, using continuous terms (percentiles) for novelty and conventionality instead of quintiles. Model (1) uses only novelty and conventionality percentiles, Model (2) adds controls, including for journal, and Model (3) adds the interaction term between novelty and conventionality. Standard errors are heteroscedasticity-robust. The models vary in the controls they use, which are noted at the bottom of the table.*

	Dependent variable:is_accepted		
	No controls	+ Controls	+ Interaction
	(1)	(2)	(3)
Novelty percentile	0.00068***	0.00123***	0.00170***
	(0.00018)	(0.00017)	(0.00027)
Conventionality percentile	0.00248***	0.00258***	0.00305***
	(0.00017)	(0.00021)	(0.00030)
Novelty pct X Conventionality pct			-0.00001*
			(0.00000)
Num. authors		0.00156***	0.00156***
		(0.00023)	(0.00023)
Num. unique journals ref'd (std.)		-0.04159***	-0.04076***
		(0.00521)	(0.00522)
Num. references		-0.00162***	-0.00159***
		(0.00024)	(0.00024)
Journal	No	Yes	Yes
Year submitted	No	Yes	Yes

Observations	27,545	27,545	27,545
R^2	0.01343	0.11512	0.11527
Adjusted R^2	0.01336	0.11325	0.11337
Residual Std. Error	0.48762 (df=27542)	0.46228 (df=27486)	0.46225 (df=27485)
F Statistic	189.98286*** (df=2; 27542)	129.71522*** (df=58; 27486)	132.45480*** (df=59; 27485)
Note:			*p<0.05; **p<0.01; ***p<0.001

Table S11. *OLS regressions predicting positive outcomes across the evaluation pipeline. Models (1)-(3) use the pooled dataset, for the (1) Desk decision, (2) Peer review recommendation, and (3) Final decision controlling for peer reviewer enthusiasm. Models (4)-(6) show analogous estimates for* Cell*, and Models (8)-(10) for* IOP *journals. For* Cell Reports*, we show only the desk decision (Model (7)) because peer review recommendations for this journal were not available. For all journals, models for outcomes other than peer reviewer recommendations use heteroscedasticity-robust standard errors, while the ones for peer reviewer recommendations are clustered at the manuscript level.*

	Pooled desk	Pooled peer review	Pooled final	Cell desk	Cell peer review	Cell final	Cell Reports desk	IOP desk	IOP peer review	IOP final
	(1)	(2)	(3)	(4)	(5)	(6)	(7)	(8)	(9)	(10)
novelty 2nd quintile	-0.018	-0.017	0.006	0.033	-0.011	0.048*	0.002	-0.037	0.007	0.006
	(0.010)	(0.012)	(0.013)	(0.018)	(0.017)	(0.023)	(0.015)	(0.019)	(0.015)	(0.013)
novelty 3rd quintile	-0.022	-0.008	0.034*	0.054**	0.014	0.140***	-0.009	-0.030	0.006	-0.004
	(0.012)	(0.014)	(0.016)	(0.020)	(0.020)	(0.027)	(0.017)	(0.021)	(0.017)	(0.015)
novelty 4th quintile	-0.005	0.005	0.038*	0.069**	0.014	0.136***	0.008	-0.008	0.048**	0.005
	(0.013)	(0.015)	(0.017)	(0.022)	(0.022)	(0.030)	(0.019)	(0.023)	(0.018)	(0.016)
novelty 5th quintile	0.056***	0.006	0.070***	0.145***	0.009	0.181***	0.045*	0.040	0.058**	0.023
	(0.013)	(0.015)	(0.017)	(0.023)	(0.023)	(0.031)	(0.020)	(0.024)	(0.019)	(0.017)

conventionality 2nd quintile	0.074***	-0.001	-0.006	-0.023	-0.035	-0.031	0.079***	0.046**	0.030*	0.007
	(0.010)	(0.013)	(0.013)	(0.018)	(0.020)	(0.025)	(0.014)	(0.017)	(0.015)	(0.013)
conventionality 3rd quintile	0.120***	0.030*	0.007	-0.035	-0.016	-0.034	0.124***	0.054**	0.040*	0.023
	(0.012)	(0.014)	(0.014)	(0.021)	(0.022)	(0.029)	(0.017)	(0.019)	(0.016)	(0.014)
conventionality 4th quintile	0.148***	0.027	0.008	-0.039	-0.044	-0.034	0.124***	0.126***	0.062***	0.022
	(0.013)	(0.016)	(0.017)	(0.025)	(0.026)	(0.033)	(0.020)	(0.022)	(0.018)	(0.016)
conventionality 5th quintile	0.146***	0.023	0.029	-0.102***	-0.053	0.005	0.159***	0.121***	0.057**	0.050**
	(0.016)	(0.018)	(0.020)	(0.030)	(0.030)	(0.040)	(0.025)	(0.026)	(0.021)	(0.019)
Peer reviewer enthusiasm			0.883***			0.737***				0.931***
			(0.010)			(0.019)				(0.012)
Num. authors	0.002***	-0.001	0.002**	0.004***	-0.001	0.001	0.001***	0.019***	0.009***	0.005***
	(0.000)	(0.001)	(0.001)	(0.001)	(0.001)	(0.001)	(0.000)	(0.002)	(0.002)	(0.001)
Num. unique journals ref'd (std.)	-0.058***	-0.015*	-0.014	-0.084***	-0.025**	-0.049***	-0.037***	-0.020*	-0.011	0.015*
	(0.005)	(0.006)	(0.007)	(0.009)	(0.009)	(0.012)	(0.007)	(0.010)	(0.008)	(0.007)
Num. references	-0.001***	0.000	-0.000	-0.001***	0.000	0.000	-0.002***	-0.004***	-0.003***	-0.003***
	(0.000)	(0.000)	(0.000)	(0.000)	(0.000)	(0.000)	(0.000)	(0.000)	(0.000)	(0.000)

Journal	Yes	Yes	Yes	Yes	Yes	Yes	Yes	Yes	Yes	Yes
Year submitted	Yes	Yes	Yes	Yes	Yes	Yes	Yes	No	No	No
Keywords	No	No	No	Yes	Yes	Yes	Yes	No	No	No
Last author pubs	No	No	No	Yes	Yes	Yes	Yes	No	No	No
Last author pubs in journal	No	No	No	Yes	Yes	Yes	Yes	No	No	No
Num. refs to journal	No	No	No	Yes	Yes	Yes	Yes	No	No	No
Manuscript type	No	No	No	No	No	No	No	Yes	Yes	Yes
Observations	27,523	21,771	8,084	8,657	12,793	3,861	12,103	6,763	8,978	4,223
R^2	0.099	0.122	0.466	0.168	0.028	0.342	0.213	0.189	0.034	0.650
Adjusted R^2	0.097	0.120	0.462	0.165	0.025	0.336	0.210	0.183	0.028	0.645
Residual Std. Error	0.472 (df=27458)	0.457 (df=21707)	0.344 (df=8019)	0.455 (df=8624)	0.493 (df=12760)	0.405 (df=3827)	0.440 (df=12057)	0.427 (df=6705)	0.390 (df=8920)	0.248 (df=4164)
F Statistic	138.948*** (df=64; 27458)	201.631*** (df=63; 21707)	209.377*** (df=64; 8019)	74.418*** (df=32; 8624)	9.648*** (df=32; 12760)	105.963*** (df=33; 3827)	102.855*** (df=45; 12057)	27.485*** (df=57; 6705)	26.316*** (df=57; 8920)	133.280*** (df=58; 4164)

Note: *p<0.05; **p<0.01; ***p<0.001

Table S12. *OLS regressions predicting positive outcomes across the evaluation pipeline for the Pooled dataset. Models (1)-(2) use Desk decision as the outcome, with and without controls respectively, and analogously for Models (3)-(4) with peer review recommendation as the outcome, and Models (5)-(6) with final decision as the outcome Models (1)-(2) and (5)-(6) use heteroscedasticity-robust standard errors, while (3)-(4) use standard errors clustered at the manuscript level.*

	Desk: No controls	Desk: + Controls + Interaction	Peer review: No controls	Peer review: + Controls + Interaction	Final: No controls	Final: + Controls + Interaction
	(1)	(2)	(3)	(4)	(5)	(6)
Novelty percentile	0.00084***	0.00127***	-0.00065**	0.00033	0.00011	0.00170***
	(0.00017)	(0.00028)	(0.00021)	(0.00034)	(0.00020)	(0.00037)
Conventionality percentile	0.00292***	0.00262***	-0.00027	0.00057	0.00067**	0.00117**
	(0.00017)	(0.00030)	(0.00022)	(0.00035)	(0.00020)	(0.00039)
Novelty pct X Conventionality pct		-0.00000		-0.00000		-0.00001*
		(0.00000)		(0.00000)		(0.00001)
Peer reviewer enthusiasm						0.88251***
						(0.01041)
Num. authors		0.00156***		-0.00088		0.00219**

		(0.00027)		(0.00061)		(0.00072)
Num. unique journals ref'd (std.)		-0.05214***		-0.01284*		-0.01465*
		(0.00521)		(0.00629)		(0.00718)
Num. references		-0.00100***		0.00004		-0.00029
		(0.00022)		(0.00021)		(0.00026)
Journal	No	Yes	No	Yes	No	Yes
Year submitted	No	Yes	No	Yes	No	Yes
Observations	27,523	27,523	21,771	21,771	15,368	8,084
R^2	0.01770	0.09795	0.00089	0.12187	0.00137	0.46644
Adjusted R^2	0.01763	0.09601	0.00080	0.11952	0.00124	0.46252
Residual Std. Error	0.49219 (df=27520)	0.47215 (df=27463)	0.48707 (df=21768)	0.45722 (df=21712)	0.44648 (df=15365)	0.34433 (df=8024)
F Statistic	253.05112*** (df=2; 27520)	174.47938*** (df=59; 27463)	6.50358** (df=2; 21768)	190.33307*** (df=58; 21712)	10.64215*** (df=2; 15365)	215.60745*** (df=59; 8024)
Note:						*p<0.05; **p<0.01; ***p<0.001

Table S13. *Negative binomial regressions for 1-year citations (Models (1)-(3)) and 5-year citations (Models (4)-(6)) using the Pooled dataset.*

	1-year: No controls	1-year: + Controls	1-year: + Interaction	5-year: No controls	5-year: + Controls	5-year: + Interaction
	(1)	(2)	(3)	(4)	(5)	(6)
Novelty percentile	0.00643***	0.00260***	0.00243***	0.00300**	-0.00198*	-0.00388*
	(0.00051)	(0.00040)	(0.00066)	(0.00115)	(0.00097)	(0.00169)
Conventionality percentile	0.00776***	-0.00251***	-0.00261***	0.00222	-0.00517***	-0.00735***
	(0.00054)	(0.00051)	(0.00074)	(0.00126)	(0.00121)	(0.00183)
Novelty pct X Conventionality pct			0.00001			0.00004
			(0.00001)			(0.00002)
Num. authors		0.00274***	0.00408***		0.02951***	0.02942***
		(0.00050)	(0.00065)		(0.00283)	(0.00283)
Last author pubs.					0.00093***	0.00091***
					(0.00013)	(0.00013)
Num. unique journals ref'd (std.)		-0.20083***	-0.20764***		-0.18431***	-0.19432***
		(0.01239)	(0.01235)		(0.03027)	(0.03034)
Num. references		0.00255***	0.00208***		-0.00021	-0.00036

		(0.00044)	(0.00043)		(0.00112)	(0.00112)
Year submitted		-0.14672***	-0.14698***		-0.21412***	-0.21337***
		(0.00550)	(0.00549)		(0.04031)	(0.03922)
Journal	No	Yes	Yes	No	Yes	Yes
Observations	25,155	25,155	25,155	2,481	2,481	2,481
R^2						
Adjusted R^2						
Residual Std. Error	1.00000 (df=25152)	1.00000 (df=25130)	1.00000 (df=25129)	1.00000 (df=2478)	1.00000 (df=2472)	1.00000 (df=2471)
F Statistic	(df=2; 25152)	(df=24; 25130)	(df=25; 25129)	(df=2; 2478)	(df=8; 2472)	(df=9; 2471)
Note:						$^{*}p<0.05$; $^{**}p<0.01$; $^{***}p<0.001$

Table S14. *OLS regressions predicting reviewer disagreement (standard deviation of binary recommendations in the first review round). Standard errors are heteroscedasticity-robust.*

	Dependent variable: recommendation_std	
	Cell	*IOP*
	(1)	(2)
novelty 2nd quintile	0.0252	-0.0082
	(0.0156)	(0.0167)
novelty 3rd quintile	0.0093	-0.0071
	(0.0182)	(0.0188)
novelty 4th quintile	0.0024	-0.0143
	(0.0197)	(0.0201)
novelty 5th quintile	-0.0164	-0.0218
	(0.0207)	(0.0207)
conventionality 2nd quintile	-0.0104	-0.0279
	(0.0174)	(0.0164)
conventionality 3rd quintile	-0.0146	-0.0279
	(0.0199)	(0.0175)
conventionality 4th quintile	-0.0228	-0.0278
	(0.0227)	(0.0193)
conventionality 5th quintile	-0.0341	-0.0372
	(0.0273)	(0.0222)
Num. authors	-0.0007	-0.0048***
	(0.0006)	(0.0014)
Num. unique journals ref'd (std.)	-0.0009	0.0136

	(0.0082)	(0.0077)
Num. references	-0.0001	0.0019^{***}
	(0.0003)	(0.0004)
Year submitted	Yes	No
Keywords	Yes	No
Last author pubs	Yes	No
Last author pubs in journal	Yes	No
Manuscript type	No	Yes
Num. refs to journal	Yes	No
Observations	3,854	3,218
R^2	0.0085	0.0130
Adjusted R^2	0.0002	0.0097
Residual Std. Error	0.2752 (df=3821)	0.2725 (df=3206)
F Statistic	1.0496 (df=32; 3821)	101.8630^{***} (df=11; 3206)
Note:		$^{*}p<0.05$; $^{**}p<0.01$; $^{***}p<0.001$

***Table S15**. OLS regressions predicting reviewer disagreement (standard deviation of binary recommendations in the first review round) for* Cell *(Models (1)-(3))* and IOP *(Models (4)-(6)). Standard errors are heteroscedasticity-robust.*

	Dependent variable: recommendation_std					
	Cell			*IOP*		
	No controls	+ Controls	+ Interaction	No controls	+ Controls	+ Interaction
	(1)	(2)	(3)	(4)	(5)	(6)
Novelty percentile	-0.0002	-0.0001	-0.0007	-0.0001	-0.0005	-0.0008
	(0.0002)	(0.0003)	(0.0005)	(0.0002)	(0.0003)	(0.0005)
Conventionality percentile	-0.0002	-0.0002	-0.0008	-0.0004	-0.0005	-0.0008
	(0.0003)	(0.0004)	(0.0005)	(0.0002)	(0.0003)	(0.0005)
Novelty pct X Conventionality pct			0.0000			0.0000
			(0.0000)			(0.0000)
Num. authors		-0.0007	-0.0007		-0.0066***	-0.0066***
		(0.0006)	(0.0006)		(0.0016)	(0.0016)
Num. unique journals ref'd (std.)		0.0026	0.0014		0.0101	0.0096
		(0.0085)	(0.0085)		(0.0090)	(0.0090)
Num. references		-0.0000	-0.0000		0.0019***	0.0019***

		(0.0003)	(0.0003)		(0.0004)	(0.0004)
Journal	No	Yes	Yes	No	Yes	Yes
Year submitted	n/a	n/a	n/a	No	Yes	Yes
Keywords	No	Yes	Yes	No	No	No
Last author pubs	No	Yes	Yes	No	No	No
Last author pubs in journal	No	Yes	Yes	No	No	No
Manuscript type	No	No	No	No	Yes	Yes
Num. refs to journal	No	Yes	Yes	No	No	No
Observations	3,854	3,854	3,854	3,218	3,218	3,218
R^2	0.0002	0.0063	0.0068	0.0015	0.0380	0.0382
Adjusted R^2	-0.0003	-0.0004	-0.0002	0.0009	0.0225	0.0224
Residual Std. Error	0.2752 (df=3851)	0.2752 (df=3827)	0.2752 (df=3826)	0.2737 (df=3215)	0.2708 (df=3166)	0.2708 (df=3165)
F Statistic	0.3686 (df =2; 3851)	0.9502 (df =26; 3827)	0.9876 (df =27; 3826)	2.4996 (df =2; 3215)	2990.4275*** (df=51; 3166)	6397.3591*** (df=52; 3165)
Note:						*p<0.05; **p<0.01; ***p<0.001

Table S16. *OLS regressions predicting Acceptance at* Cell *using novelty and conventionality calculated at* submission*, as opposed to publication. Standard errors are heteroscedasticity-robust.*

	Dependent variable:is_accepted		
	No controls	+ Controls	+ Interaction
	(1)	(2)	(3)
Novelty percentile	0.00085	0.00205**	0.00159
	(0.00044)	(0.00069)	(0.00109)
Conventionality percentile	0.00130**	0.00148*	0.00106
	(0.00042)	(0.00074)	(0.00108)
Novelty pct X Conventionality pct			0.00001
			(0.00002)
Num. authors		0.00287	0.00287
		(0.00188)	(0.00188)
Num. unique journals ref'd (std.)		-0.00011	0.00047
		(0.01596)	(0.01595)
Num. references		0.00209**	0.00211**
		(0.00069)	(0.00069)
Journal	No	Yes	Yes
Year submitted	No	Yes	Yes
Keywords	No	Yes	Yes
Last author pubs	No	Yes	Yes

Last author pubs in *Cell*	No	Yes	Yes
Num. refs to *Cell*	No	Yes	Yes
Observations	2,466	1,314	1,314
R^2	0.00405	0.20326	0.20343
Adjusted R^2	0.00324	0.18905	0.18859
Residual Std. Error	0.36737 (df=2463)	0.41396 (df=1290)	0.41407 (df=1289)
F Statistic	5.12316^{**} (df=2; 2463)	35.85604^{***} (df=23; 1290)	34.33424^{***} (df=24; 1289)
Note:			$^{*}p<0.05$; $^{**}p<0.01$; $^{***}p<0.001$

Table S17*. Log-odd ratios from logistic regressions predicting Acceptance in the Pooled sample,* Cell*,* Cell Reports*, and* IOP *journals. To avoid convergence errors in estimation of the Physics journals model, journals with fewer than 100 complete observations or no variation in the outcome are excluded.*

	Dependent variable: is_accepted			
	Pooled	*Cell*	*Cell Reports*	*IOP*
	(1)	(2)	(3)	(4)
novelty 2nd quintile	-0.089	0.208*	-0.004	-0.067
	(0.048)	(0.098)	(0.079)	(0.108)
novelty 3rd quintile	-0.078	0.598***	-0.016	-0.057
	(0.055)	(0.112)	(0.090)	(0.122)
novelty 4th quintile	-0.008	0.647***	0.040	0.183
	(0.060)	(0.125)	(0.101)	(0.131)
novelty 5th quintile	0.283***	1.036***	0.305**	0.457***
	(0.063)	(0.128)	(0.109)	(0.136)
conventionality 2nd quintile	0.340***	-0.245*	0.336***	0.249*
	(0.048)	(0.106)	(0.077)	(0.100)
conventionality 3rd quintile	0.596***	-0.254*	0.476***	0.439***
	(0.056)	(0.121)	(0.093)	(0.112)
conventionality 4th quintile	0.707***	-0.303*	0.442***	0.811***
	(0.064)	(0.141)	(0.108)	(0.130)
conventionality 5th quintile	0.819***	-0.464**	0.710***	0.868***
	(0.077)	(0.169)	(0.131)	(0.154)
Num. authors	0.029***	0.008*	0.041***	0.132***
	(0.002)	(0.003)	(0.004)	(0.012)

Num. unique journals ref'd (std.)	-0.219***	-0.485***	-0.248***	-0.037
	(0.024)	(0.052)	(0.040)	(0.058)
Num. references	-0.007***	-0.002	-0.021***	-0.037***
	(0.001)	(0.002)	(0.002)	(0.003)
Journal	Yes	No	No	Yes
Year submitted	Yes	Yes	Yes	No
Keywords	No	Yes	Yes	No
Last author pubs	No	Yes	Yes	No
Last author pubs in journal	No	Yes	Yes	No
Manuscript type	No	No	No	Yes
Num. refs to journal	No	Yes	Yes	No
Observations	26,334	8,657	12,103	5,574
R^2				
Adjusted R^2				
Residual Std. Error	1.000 (df=26299)	1.000 (df=8624)	1.000 (df=12057)	1.000 (df=5546)
F Statistic	(df=34; 26299)	(df=32; 8624)	(df=45; 12057)	(df=27; 5546)
Note:				*p<0.05; **p<0.01; ***p<0.001

5. Supplementary references

Bornmann, L., Tekles, A., Zhang, H. H., & Ye, F. Y. (2019). Do we measure novelty when we analyze unusual combinations of cited references? A validation study of bibliometric novelty indicators based on F1000Prime data. Journal of Informetrics, 13(4), 100979. https://doi.org/10.1016/j.joi.2019.100979

Introduction—GROBID Documentation. (n.d.). Retrieved May 14, 2021, from https://grobid.readthedocs.io/en/latest/Introduction/

Lee, Y.-N., Walsh, J. P., & Wang, J. (2015). Creativity in scientific teams: Unpacking novelty and impact. Research Policy, 44(3), 684–697. https://doi.org/10.1016/j.respol.2014.10.007

Uzzi, B., Mukherjee, S., Stringer, M., & Jones, B. (2013). Atypical Combinations and Scientific Impact. Science, 342(6157), 468–472. https://doi.org/10.1126/science.1240474

Visser, M., van Eck, N. J., & Waltman, L. (2021). Large-scale comparison of bibliographic data sources: Scopus, Web of Science, Dimensions, Crossref, and Microsoft Academic. Quantitative Science Studies, 2(1), 20–41. https://doi.org/10.1162/qss_a_00112
